# What is the quality of care at the end of life? Qualitative findings from a nationally-representative post-bereavement survey across England and Wales

**DOI:** 10.1177/13558196251398678

**Published:** 2025-12-29

**Authors:** Joanna Goodrich, Sophie Pask, Chukwuebuka Okwuosa, Therese Johansson, Lynn Laidlaw, Cara Ghiglieri, Rachel Chambers, Anna E. Bone, Stephen Barclay, Fliss E. M. Murtagh, Katherine E. Sleeman

**Affiliations:** 1 Cicely Saunders Institute of Palliative Care, Policy and Rehabilitation, 4616 King’s College London, London, UK; 2 Wolfson Palliative Care Research Centre, 12195 Hull York Medical School, University of Hull, Hull, UK; 3 Palliative & End of Life Care in Cambridge (PELiCAM), Primary Care Unit, Department of Public Health and Primary Care, 151912 University of Cambridge, Cambridge, UK

**Keywords:** health service quality, palliative care, end of life care, patient experience, family carers

## Abstract

**Objectives:**

To explore the quality of end-of-life care in England and Wales using the experiences of bereaved family carers, and to develop person-centred quality of care domains for end-of-life care.

**Methods:**

Qualitative analysis of free-text responses from a nationally-representative cross-sectional post-bereavement survey. Inductive thematic analysis of free-text responses to open-ended questions about care in last 3 months of life, circumstances of death, and experiences of care and bereavement, guided by the Institute of Medicine’s quality domains. Participants were adults who registered the death of an adult relative in England and Wales between August and December 2022, identified using mortality data and stratified sampling (by age, gender, cause of death, place of death and geographical area).

**Results:**

Of 1194 respondents, 1083 (90.7%) gave at least one free-text response. Six themes about quality of end-of-life care were identified: (1) accessing care; (2) timely and coordinated care; (3) individualised care; (4) the nature of communication and care; (5) family-centred care and support; and (6) safe and equitable care. Difficulty accessing care, challenges navigating a complex system, and poorly-coordinated care were interpreted as leading to a lack of physical and psychological safety. Timeliness of care was considered paramount but often not achieved. *How* care was provided was as important as *what* was provided: empathic relational care (in contrast to transactional, task-based care) led to dying people and their families reporting feeling reassured, supported and safe.

**Conclusions:**

We identify aspects of quality important for care which are currently not always achieved, and provide a refined model of the quality of end-of-life care to guide policy and research.

## Introduction

Internationally, the challenge of ensuring high quality care towards the end of life remains a public and political priority.^1,2^ In England and Wales, end-of-life care is provided through a combination of specialist palliative care services (delivered in hospices, hospitals and the community) and by non-specialist services (delivered in community and hospital settings by a combination of primary health care services (GPs and community nurses) and hospital staff, respectively). In cross-country comparisons of the quality of end-of-life care, the UK has consistently been placed at the top of a global league table.^3^ However, complaints to the UK’s Parliamentary and Health Services Ombudsman have shown that poor care towards the end of life is a common and recurring theme amongst those complaints.^4^

How quality of health care is understood and measured shapes the way care is delivered and experienced.^5^ Societal influences (e.g., changing population demographics, growing demand for care, increased multimorbidity, and lack of resources in health care), and the expectations and interests of different stakeholders, all influence the debate about quality of care.^6^ The Institute of Medicine’s report ‘Crossing the Quality Chasm’ conceptualised quality of health care as six domains: safety, timeliness, effectiveness, efficiency, equity and patient-centredness.^7^ These domains (or variants of them) are widely used in monitoring and improving health services.^8^ However, aspects of quality of particular importance at the end of life are not included, such as system navigation and access to care.^6^

In many countries, including the UK, there has been a sustained increase in home deaths since the Covid 19 pandemic.^9^ Shifting care from the acute hospital setting into the community is a policy priority for many countries.^10^ Increasing austerity and widening inequalities, and growing pressures on health and social care services, have led to widespread concern about the quality of care for people approaching the end of life.^11^ Family carers provide up to 90% of home-based end-of-life care^12^ and are in a unique position to provide inclusive overview of the quality and experience of end-of-life care. Our recent nationally representative post-bereavement survey in England and Wales, found almost half of the participants were unhappy with some aspects of the care received by the person who died.^13^

Our aim in this study was to explore the quality of end-of-life care in England and Wales using the experiences of bereaved family carers, and to develop person-centred quality of care domains for end-of-life care.

## Methods

### Study design

This paper comprises qualitative analysis of free-text responses to open-ended questions from a nationally-representative post-bereavement cross-sectional survey across England and Wales. A report of findings from the survey (and further detail on the methodology) has been published elsewhere.^13^

We used a modified version of the QUALYCARE survey, a validated questionnaire to measure bereaved relatives’ care experiences of the last months of life.^14^

The survey comprised closed and open-ended questions about aspects of health care quality: the care respondents’ relatives received in the last 3 months and 1 week of life, the circumstances of their relative’s death, and their own experiences of care and bereavement (Table 1).

**Table 1.** Open-ended questions.

Section 2A	**Care received in your relative's last 3 months of life**.• Did your relative have contacts with a palliative care team during their last 3 months of life?
	*• Please feel free to comment on the support received (or not received) from the palliative care team*
	*• Were there any services that you or your relatives found difficult to access or were unable to access? Please name the services and reasons for the difficulty*
Section 2B	Your assessment of care received during your relative’s last 3 months of life
	• *Please feel free to write details about aspects of the care that you were unhappy with here or in the blank sheet at the end of the questionnaire*
Section 3	**The last week before your relative died**
	• *If any, what were your relative’s main problems or concerns in the last week before they died? Please write down*
Section 4	**Circumstances of death and personal preferences**
	Did any health or care professional talk to you, other family members, or friends about the fact that [your relative] might die?
	• *Please feel free to comment how you feel about the way this was discussed (or not discussed)*
	Did [your relative] discuss their preference about where they wanted to die with any health or care professional?
	• *If yes, please feel free to comment how you feel about the way this was discussed (or not discussed)*
	Did their preference change at any time during the last 3 months?
	• *If yes, in what ways did it change and why do you think that might have been?*
	• *Please feel free to make any comments about choices and preferences related to where they died and the circumstances surrounding their death*
Section 6	**Final question**
	• *If there is anything else you would like to tell us about any aspect of the care received or care you would have liked to receive please use the space below*

### Identification of survey respondents

Survey respondents were adults (aged ≥18) who had registered the death of an adult family member 6–10 months before survey distribution. The timeframe was chosen to avoid the immediate bereavement period and the anniversary of the death.^15^ Sudden deaths (caused by accidents, suicide, or homicide), deaths registered by non-relatives and deaths subject to inquest were excluded (see Sampling Frame, Table S1, Online Supplement for included causes of death).

Deaths were identified using Office for National Statistics (ONS) mortality data. A representative sample across England and Wales was sought using stratified sampling approach, according to the deceased’s gender, cause of death, place of death, age and geographical area. Surveys were sent out to family members of the identified decedents.

### Data collection

The Office for National Statistics (ONS) administered the survey to 3000 people (1500 in England and 1500 in Wales). Data were collected between May and November 2023 for deaths that occurred between August and December 2022. Prospective respondents were sent a paper copy of the survey with a pre-paid return envelope but could complete it online or via telephone if preferred. Respondents could opt out by returning a pre-paid slip to the ONS or contacting the research team. If no response was received, the initial invitation to take part was followed by two reminder letters. The final reminder letter included another paper copy of the survey.

### Data analysis

All data from respondents who provided at least one free-text response to any of the open-ended questions were included. Responses ranged from brief clarifying points to several pages explaining experiences in detail. The free text was analysed using Braun and Clarke’s reflexive thematic analysis^16^ by a team of researchers (JG, SP and CO). Although we were guided by the Institute of Medicine’s commonly adopted domains of quality,^7^ patterned meaning in relation to ‘quality of care’ and meaning of quality expressed by respondents were inductively sought to augment or challenge existing definitions of quality. Data were coded using semantic (i.e., descriptive) and latent (i.e., implicit meaning) codes. We broadly adopted thematic analysis as a ‘realist’ method, treating what people said as reflecting their views and perspectives.^16^ Initial themes were generated by compiling clusters of codes that shared a core concept, and were critically reviewed in research team discussions. Free-text responses were managed in NVivo and demographic data were analysed using IBM SPSS [version 28]. We suppressed demographic data where counts were less than 3 to ensure that respondents could not be identified.

### Patient and public involvement

The study was guided by six Patient and Public Involvement (PPI) partners with lived experience of advanced illness or caring for a family member at the end of life. They contributed to study design, providing advice on sensitively introducing the survey, and suggesting amendments to simplify the opt-out process. They helped generate initial themes and refine the themes and subthemes. Regular meetings between researchers and two PPI partners during the analysis stage enabled reflexive discussion. (See Online Supplement 1, (GRIPP2 checklist) for PPI partners’ reflections on their involvement).

### Ethical approval

This study received ethical approval from King’s College London Research Ethics Committee (HR/DP-21/22-24690).

The survey was sent out with an accompanying study information letter explaining the study aim, potential risks of distress, how data would be used, that data would be anonymised, that participation was voluntary and how to opt out. Consent to use data was assumed for surveys received from respondents. All those approached received bereavement support information and helpline numbers together with their study invitation.

## Results

A total of 1194 surveys were returned (response rate 39.8%): 1071 paper surveys, 112 online, and 11 by telephone. Of the 1194 respondents, 1083 (90.7%) gave at least one free-text response. Of these, the mean age was 63.1 (SD = 11.9), 696 (64.3%) were female, 611 (56.4%) were the child of the person who died, and 822 (75.9%) cared for the decedent during their last 3 months of life. Most completed the paper survey (*n* = 975, 90.0%), with some completing online (*n* = 97, 9.0%) or over the phone (*n* = 11, 1.0%). Table S2, Online Supplement presents the demographic characteristics of the decedents about whom the respondents were surveyed.

Six themes were identified in relation to the quality of care for those with advanced illness and their family carers. Quotes from participants to illustrate these themes are provided in Table 2.

**Table 2.** Quotes from participants.

		Quotation No.
Theme 1: Accessing care		
Navigating the system	“It makes me realise how cross I am at the lack of support we had. We had to ask and push for everything and in a situation which we had never been in before.” (Female, 78, Wales)	1
	“When my husband required continuing health care [funding], I had to find out who to speak to and how to access [funding and care]. It is arbitrary over who gets access to it and who doesn’t. I’ve spoken to other people who care for their families and they don’t know what care is accessible/available to them. There needs to be a course/education provided to family members about what care they have access to.” (Female, 71, England)	2
Access to professionals and services	“My father was housebound, my mother was his full-time carer. There was NO support from the GP or anyone at that practice. They refused a home visit. My father had not seen a GP for 3 YEARS before he died.” (Female, 52, Wales)	3
	“GP services – [it was] difficult to get an appointment face to face, difficult to get advice, difficult having to deal with different personnel every time - All of this added to the immense stress we felt as family exhausted from caring for and supporting Dad, stressful to him too.” (Female, 59, Wales)	4
	“In the night just a couple of days before my relative’s passing, it was very difficult to reach the district nurses to come over and administer more medication. We waited 8 h without any response. My relative was in a state of extreme pain, stress and agitation and we were told by the district nurses that no-one would be able to help despite my relative being an ‘urgent case’ patient. The hospice-at-home nurses were unable to provide a call-out nurse as they had no available staff. Phone calls were made between 2am and 6am. The district nurse had received no information that we had been calling since 2am.” (Female, 60, England)	5
	“Local pharmacy didn’t always have stock of meds. Weekend opening times of pharmacy could be problematic. These two points resulted in me having to travel 20 miles to pick up prescriptions and medications which deprived me of precious time with my husband. Plus caused added stress having to find someone to sit with him. My husband was very anxious with this situation.” (Female, 69, Wales)	6
Theme 2: Timely and coordinated care		
Timeliness of care	“Waiting for carers to be organised to care for him at home before he could come out of hospital [….] very unhelpful. They were unable to organise the needed package and unable to give a time frame on when they could be sorted. For a man with only weeks left to live - that was not helpful. Spending his last days at home was his wish but making this possible was very difficult and almost didn’t happen.” (Female, 49, England)	7
	“Palliative care was good when we finally got somebody, as nobody would listen to me...but it was a bit late”. (Female, 55, England)	8
	“Often needed professional help at night due to his inability to remain in a good position for comfort to sleep - sometimes a bit of a wait but not too severe - 111 doctors on duty excellent- short waits most times reassuring for me too as an older carer!” (Female, 84, England)	9
	“It was difficult waiting for the district nurses to come and inject him, the waiting was unbearable.” (Female,71, England)	10
Coordination of care	“Everything felt like a battle and meanwhile I was supposed to be caring for my sister and spending precious time with her, not chasing for help.” (Female, 61, Wales)	11
	“While working full-time I had coordinated all health appointments, care provision and emergencies. Communication between the relevant organisations was poor or non-existent.” (Female, 61, England)	12
	“In the last 3 months of my uncle’s life, there were many people from many departments contacting us. We (I) had to relay the information again and again. There seemed to be no coordination of activity unless I pushed and pushed. This was extremely hard on my uncle and also myself, at a very difficult time. Incorrect information, things not followed up, no help available etc- we felt so very much alone and adrift.” (Female, 47, Wales)	13
Theme 3: Individualised care		
Response to needs	“She was also in obvious mental and physical pain. […] The community palliative care were incredible - caring, thoughtful, and helped my mother in many ways. Without them, her death would have been more painful and she would have endured more suffering.” (Female, 39, England)	14
	“My father did not feel listened to. He had multiple health issues but only one or two would be prioritised and what he felt were his main problems were dismissed. His symptoms were unusual and my father experienced constant pain [which] most of the Drs dismissed to treat other problems”. (Female, 44, England)	15
	“Mum’s final weeks were distressing and she was quite unhappy. No account seemed to be taken of her psychological and mental wellbeing needs when placement was decided. […] There seemed to be a gap in the nursing home’s capacity to absorb and address this… […] However, this was also an issue during her hospital care. Again, emphasis post-stroke was on physical care, but lacked holistic approach that would have ensured psychological needs were properly met.” (Male, 64, England)	16
	“My wife had a hearing problem but no one wanted to help. She would tell the carers on many occasions. Also, she could not swallow the hard pills and capsules. They did not offer help other than keep instructing her. In her frail state, she needed better help and understanding.” (Male, 74, England)	17
	“More care needs to be given to dementia patients. There is not enough education or understanding around the illness. NHS staff need to be dementia aware and show more compassion and care. The only people on my dad’s journey that were kind and understanding were the ones who have had a relative with dementia. More info [needs to be] given to understand what is involved with end-of-life care. Not a nice experience which will haunt me for the rest of my life.” (Female, 50, Wales)	18
Preferences about place of death	“All services were very difficult. My mother’s ‘strongest’ wish was to remain at home! I struggled to get help with everything until the last 48 h of her life when finally the district nurses helped.” (Female, 60, Wales)	19
	“I feel the care we received from the GP on [date] was excellent. He was kind and compassionate, and we tried to include my dad in our decision for him to stay in his own bed and not have the trauma of going into hospital.” (Female, 60, Wales)	20
	“He always asked me that he could die at home but after the way I had to care for him and the pain getting worse he wanted to go into hospice. After he was admitted, his mood lifted he looked so much happier.” (Female, 59, Wales)	21
	“My father died on a busy ward with a very noisy and disruptive man in the bed next to us. Although the curtains were closed it was not a peaceful, respectful or appropriate way to end his life.” (Female, 41, England)	22
Theme 4: The nature of communication and care		
Communicating with family carers	“Living 2 hours away we found it very difficult to get any updates while mum was both in ITU and [then] on a ward. If the phone was answered at all no-one seemed to know what was going on. Even when we went into the hospital to see her and asked what was happening, we were told different information by different people. Staff did not have time to talk to us.” (Female, 47, Wales)	23
	“We found his last days in hospital were distressing…. We found it difficult to find the wards he was being moved to as the telephone was never being answered. We had to travel several miles to get information. This was the hardest part of his stay in hospital up to his death.” (Female, 82, Wales)	24
	“I believe having nursed mum at our own home for over a year and seen her though 2 ‘end of life’ episodes with the support of community team, local GP, supporting healthcare professionals, our own family GP, we built up a bank of information. Knowledge we were able to draw on when in discussion with the care home, to help make suggestions + positive inputs into her care plan.” (Female, 69, England)	25
Honest conversations	“They were very professional and polite and listened to my questions and gave me honest answers. They made the ordeal more bearable.” (Male, 55, England)	26
	“I wished that I had known that her health had changed during the week. I would have spent time with her…I wish I would have done this for my mother- at least to have said goodbye, to have some warning, when clearly the care home staff knew she was at end of life, but didn’t phone to say.” (Female, 57, Wales)	27
Relational approach	“The hospital doctor, who all along was difficult to reach, invited me in a room and, without preamble, said that they would not be resuscitating my mother. His blatant agenda was quite shocking, along with his cold, detached manner…” (Female, 68, England)	28
	“The care staff at my Mum’s residential care home were incredible. They were diligent, supportive of us and of Mum, professional, compassionate and loving. We felt as if we were surrounded by close family. I feel deep appreciation of their support and hard work through that difficult time.” (Female, 62, England)	29
Theme 5: Family-centred care and support		
The challenges and demands of caring	“My brother sacrificed 4 years of his life and eventually lost his income [to care for our mother]… Mum wanted to remain in her own home but she needed 24/7 care. The medical profession, being under severe pressure were not able to offer my mum and her carers the support they needed. I am convinced that the impact of [his] sacrifice will continue to have detrimental effects for years to come.” (Female, 60, England)	30
Support for family carers	“We were struggling with finding anyone to spend time with our mum so that we could have a break. We were either working or caring and we needed someone to be able to sit with mum for a couple of hours. We never got this help.” (Female, 53, Wales)	31
	“Our ‘hospice-at-home’ support was amazing. They sorted everything and treated my Dad with dignity. Any request was supported. They were also a huge help to me. In particular the night sitting service was a huge help when I was exhausted. I could have hugged the nurse when she arrived.” (Female, 38, England)	32
Post-bereavement support	“Something should be in place where you can go to get advice of what you need to do regarding arrangements and getting support when you feel overwhelmed with what needs to be done.” (Female, 56, England)	33
Theme 6: Safe and equitable care		
Emotional and physical safety	“It is quite shocking that a 97-year-old man should have to carry the sole burden of a 90-year-old wife with Alzheimer’s”. (Female, 66, England)	34
	“I don’t believe my brother needed physical care but emotional care - the services offered were not suitable or frequent enough to build the relationship needed for end of life, after treatment stopped. He became scared and felt very disregarded.” (Female, 52, Wales)	35
	“I felt there was a lack of support for myself. Being in full time employment, dependent on one salary in the household! I felt the pressure as a carer overwhelming, affecting the relationship as I evolved [to] becoming a carer not a daughter. I found community support lacking, particularly services, in the provision of activities, which would have benefited mum and would have provided support to myself as a carer […] Ultimately I felt on a very lonely journey with many decisions having to be made and in the main [unsure] whether they were the right ones.” (Female, 57, Wales)	36
	“GP was very good at explaining but once end-of-life care was decided there was no more visits”. (Female, 62, England)	37
	“Despite repeated phone calls from the family (me) and the nurses at the care home medications were not prescribed correctly thus not administered to relieve pain at the end of my mum’s life…” (Female, 63, England)	38
Equitable care	“Nothing available at all in our area when it was desperately needed” (Female, 45, Wales)	39
	“My mother had cancer, we know this was terminal and there was an amazing support network in place. My father, now 90, is ill due to old age - very little support - this is much harder to cope with.” (Female, 61, Wales)	40
	“I believe if you get to 88 years old you are not high on the Hospital agenda.” (Male, 70, England)	41
	“We had to pay private carers. Fortunately, we could afford this. Otherwise I could not have coped. I am very concerned about people who cannot afford private care.” (Male, 84, Wales)	42

### Accessing care

Accessing care was fundamental to the quality of care experienced. When care could not be accessed, this was described as ‘falling at the first hurdle’ and impacted on all other aspects of quality.

#### Navigating the system

Many reported difficulties in navigating a complex care system, having to *‘ask and push for everything in a situation *[they]* had never been in before*.' (Female, 78, Wales). Trying to understand what help was available, and where and how to access it, was difficult and frustrating (Quote (Q)1).

A key challenge for many related to identifying potential financial support and navigating the application processes for these funds (Q2). In many cases, private care was sought as an alternative.

#### Access to professionals and services

There were difficulties with getting ‘into the system’, for example getting advice or assessments by professionals, accessing a care package (i.e., help from carers from social services coming into the home), or finding a suitable care home with available beds.

Access to health and care professionals, particularly in the community, was frequently reported as difficult and stressful. There was often a clear mismatch between what was expected and what was experienced, especially in relation to obtaining a face-to-face appointment or home visit. This increased carers’ emotional work, which was expressed with feelings of surprise, shock, disrespect and abandonment (Q3, Q4).

Palliative care services were not always offered or available. Many described how once their relative got referred to palliative care, their situation improved; but eligibility for palliative care was not clear to respondents.

Access to care was hampered by geographical distance and limited transport. Challenges were often reported ‘out-of-hours’ (i.e. evenings, weekends, and public holidays) (Q5). This led to feelings of ‘*isolation and abandonment’* and that ‘*no-one really cares.'* (Female, 65, England).

Finding their way around the system to obtain end-of life-medicines was often reported as difficult, particularly out-of-hours, not knowing how to get medications prescribed, which pharmacies stocked what they needed and their opening hours. These challenges created additional stress for everyone (Q6).

### Timely and coordinated care

Delays in receiving care, and poorly coordinated care, took on added significance as the remaining time family carers had with their relative was precious. Family carers did not want to spend this time chasing and coordinating care, but felt they had no choice but to do so.

#### Timeliness of care

Family carers very often mentioned ‘*waiting for everything’,* while at the same time having an acute sense that time was running out. This included waiting for appointments, tests, test results, and treatments, with frustration about the ‘*snail’s pace at which diagnostic tests, scans and appointments are scheduled.'* (Female, 72, Wales). There were frequent examples of delays in being allocated beds in hospitals, care homes or hospices, and accessing support services (Q7). Often the response to a person’s care needs came too late which increased suffering. This included provision of palliative care (Q8).

Timeliness in managing symptoms, particularly pain relief, was a prominent focus. Timely responses from professionals were valued and reassuring (Q9) but delays of any length led to upsetting experiences; waiting while their family member was in pain, for an ambulance to arrive, in the emergency department, or at home for a return phone call from a professional, or a visit to administer medications was described as ‘*difficult’* and that *‘the waiting was unbearable.'* (Female, 71, England, Q10).

#### Coordination of care

Accessing services was made more challenging and time-consuming by a lack of coordination. Services were described as ‘*not joined up’* and this created additional work for family carers when ‘*everything felt like a battle.’* (Female, 61, Wales) as they took on the responsibility of coordinating care. This further detracted from meaningful time spent with the person who was ill (Q11, Q12).

Promises of care were sometimes unreliable with examples of professionals being in touch once but then not being available again. Several respondents suggested that having one point of contact or professional to coordinate care would have improved their experiences (Q13).

### Individualised care

Ensuring care was tailored to individual needs, preferences and expectations led to better experiences of care for patients and family carers. Disappointment, distress, or sometimes anger, were expressed when this was not achieved.

#### Response to needs

There were examples provided of responsive, individualised symptom management that demonstrated the positive impact care of this type had on family carers, both at the time of and after the death of their relative (Q14). However, respondents also described distressing situations when symptoms, particularly pain, were not managed well. Sometimes, when the patient had multiple conditions, symptom management was not aligned to what was important to the patient, and there were examples of where the broader, more general needs, of individuals (related to frailty rather than specific conditions) were not well met (Q14, Q15, Q16, Q17).

The end-of-life care needs of people with certain conditions, including neurological diseases, dementia and intellectual disabilities, were described as not well-understood in health care services. Family carers of people with dementia frequently reported difficulties across varied aspects of quality (Q18).

#### Preferences about place of death

Preferences about where the patient wanted to be cared for until death were often referred to by family carers, as well as the importance of honouring these wishes. Most frequently, the preference had been to stay at home, and although not always achieved (Q19), it was greatly appreciated when professionals were supportive in trying to realise these preferences (Q20).

At times, plans had to change when it became clear that family carers could not manage at home. Family carers occasionally described disappointment about this but also understood that the needs of their relative could not be met in their preferred place (Q21).

Hospital settings, including both emergency departments and wards, were often described as noisy, cramped, uncomfortable places that lacked privacy, and that it was *‘not a peaceful, respectful or appropriate way to end [someone’s] life.’* (Female, 41, England, Q22).

### The nature of communication and care

The way in which professionals care, and their communication skills, were of fundamental importance in end-of-life care. Family carers appreciated honest conversations and empathic communication where they felt listened to, informed, and involved in decisions.

#### Communicating with family carers

Family carers described information provided by professionals variously as non-existent, helpful or conflicting. Accounts were given of being given different information by different people, in hospital settings particularly, where staff were too busy to speak to relatives. Lack of information amplified anxiety and stress (Q23, Q24).

Being listened to and kept ‘in the loop’ was very important to family carers, and provided reassurance.

Shared decision-making between professionals and family carers did not always happen, but it was valued when families were involved in a meaningful way and felt their knowledge of the individual was being recognised (Q25).

#### Honest conversations

Recognising and communicating that their relative was nearing the end of life was very important to family carers. Listening to their questions and providing honest answers ‘*made the ordeal more bearable.’* (Male, 55. England, Q26).

Communication about deterioration, dying, and what to expect at the end of life, often did not happen; consequently, families felt unprepared or realised ‘too late’ that their relative was going to die, often leading to them to regret decisions they had made without complete information, or that they had not had the chance to say goodbye (Q27).

#### Relational approach

*How* communication and care were conducted were as important as *what* was said or done. Accounts were given of care ranging from rude, dismissive and uncaring, with professionals having a ‘cold detached manner’ (Female, 68, England, Q28), to compassionate, kind, gentle, and empathetic. The way in which families felt professionals communicated and cared for their relative made a profound difference. When care was *‘diligent, supportive….professional, compassionate and loving’* there was ‘*deep appreciation of* [professionals’] *support and hard work through that difficult time.’* (Female, 62, England, Q29).

### Family-centred care and support

Family carers frequently contributed to care at home, even if they did not feel competent to do so.

#### The challenges and demands of caring

Family carers often filled the gaps where professional care was not available, or it was felt to be inadequate. This had financial, emotional and health implications for family carers which could have ‘*detrimental effects for years to come.’* (Female, 60, England, Q30).

#### Support for family carers

Often, no support for family carers appeared to be available or offered. When family carers did try to seek support for themselves (including respite care), it was hard to find, but where available, it made a marked difference for everyone concerned (Q31, Q32).

The impact of the work of caring on family carers included: feelings of being alone; and guilt and regret after their relative died, especially when they felt they had not done enough to procure care and asked themselves ‘*could I have fought harder?’* (Female, 57, Wales).

#### Post-bereavement support

Family carers reported difficulties immediately after the death of their relative, with a lack of information about what to do or who to contact, or delays in getting hold of professionals, or professionals arriving to support them (Q33). Bureaucratic delays added to the stress of the family carers. Conversely, they described how both formal and informal support from professionals helped their grief process.

### Safe and equitable care

Safety was considered to be jeopardised when care that felt necessary could not be accessed. Safety was often conceptualised as emotional safety. However, incidents of physically unsafe care were also described.

#### Emotional and physical safety

Family carers frequently felt-ill equipped to provide care, in terms of confidence and competence, which led to feelings of their relative being unsafe. In many cases the main carers were themselves frail, unwell or elderly, and physically struggled to provide the care required (Q34). Reassurance was key to feeling emotionally safe for both patients and family carers (Q35). Family carers reported feeling anxious and uncertain that they were doing the right things due to the lack of practical support available (Q36).

Family carers described perceptions of diminishing care towards the end of life (when fewer clinical interventions were in place), at a time when they, as carers, especially needed the support of continued contact with health services (Q37).

Accounts of lack of physical safety were given, for example, very ill relatives being discharged home from hospital with inadequate support in place, either because home carers were unavailable (or not yet in place) or because family carers felt unconfident to carry out necessary tasks. There were also instances where their relatives were discharged from a care setting despite living on their own, which carers felt was unsafe (e.g. because of risk of falling). These safety concerns caused anxiety and stress for family carers.

Family carers also recounted doubts about the skills and expertise of some professionals and reported issues including with medication, pressure sores, falls and hospital acquired infections (Q38).

#### Equitable care

Responses reported that geographical disparity, socioeconomic differences, and variation in access due to demographic or clinical characteristics led to inequality in care. In some areas it seems there was *‘nothing available at all…when it was desperately needed.’* (Female, 45, Wales, Q39).

For some, when specialist palliative care was provided, the experience of care was positive, but high-quality specialist palliative care was not available to everybody. Disparities in end-of-life care according to condition was also noted (Q40).

For some conditions, care appeared to be less easy to find: as already shown, family carers looking after relatives with dementia reported particular difficulties in accessing support.

There was a perception that ageism underlay attitudes towards older people, when there was a poor response to requests for help or a ‘dismissive’ attitude, especially when home visits were not offered; it was felt that because of their age they were not treated equally: ‘*I believe if you get to 88 years old you are not high on the hospital agenda.'* (Male, 70, England, Q41).

Private care (e.g., home care agencies) was accessible to those able to pay and was often put in place more quickly than statutory services, though there were still instances of staff shortages in private care agencies (Q42).

## Discussion

This is the first nationally-representative study of the quality of end-of-life care in England and Wales since 2015. We provide a detailed picture of the quality of end-of-life care from the family carer perspective and identify challenges that need urgent attention. This study found that compounding problems of difficulty accessing care, challenges navigating a complex system, and poorly coordinated care can result in a perceived lack of physical and psychological safety for people who die and their families. Timeliness of care is of paramount importance when someone is dying and every day matters, but is often not achieved, adding worry to an already stressful situation. *How* care is provided, regardless of setting, is as important as *what* is provided: empathic and relational care leads to dying people and their family carers feeling reassured, supported and safe, as opposed to abandoned and alone.

While issues of inadequate community support, lack of coordinated care and untimely hospital discharge have been identified by bereaved carers before,^17^ the national sampling framework and free-text responses in this survey provide deeper insight into the quality of end-of-life care across a variety of disease groups that earlier studies have not addressed.^14^

**Figure 1.**
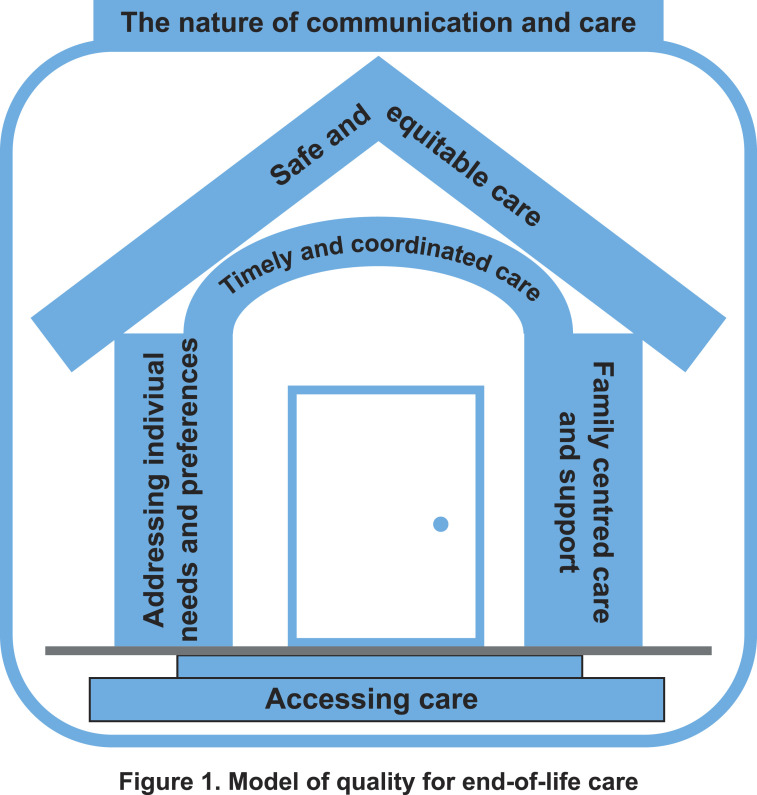
Model of quality for end-of-life care. Components of quality.

This study provides novel understanding of the essential domains of health care quality for people approaching the end of life based on the reported experiences of bereaved family carers. We have drawn on the Institute of Medicine’s quality framework^7^ to underpin and extend insights into what ‘quality’ means in end-of-life care. Together with Beattie’s additional domains,^6^ we propose a model for conceptualising quality in end-of-life care (Figure 1). This builds on the House of Care model for people with long term conditions, which emphasises the interdependency of each quality domain as well as the components needed to ensure a well-functioning, coordinated, and sustainable service delivery model.^18^ Our model is intended as a framework that reflects experiences of bereaved families. It can be used by practitioners and policy makers as the basis to produce or revise existing quality statements. These findings from a survey in England and Wales are potentially transferable to settings outside the UK where there are similarities in context and similar implications for family carers and professional community services.^9^

Accessing care, not named as a distinct quality domain in the Institute of Medicine’s framework, is fundamental and serves as the ‘steps’ into the house. System navigation is a key part of access, especially as caring for a dying person is often an unfamiliar experience.^19^ When care is not accessible, families often step in, sometimes without sufficient knowledge, support, or preparation, which risks safety and adverse consequences for both patients and their families. Therefore, family centred care and support is one of the pillars necessary to ensure safe and equitable care. Addressing individual needs and preferences is also an essential pillar to deliver high quality end-of-life care; family carers highlight the importance of managing symptoms (particularly pain) and addressing preferences for place of care. Care needs to be timely and coordinated to support safe care provision. Any delay in the context of end of life is felt intensely.

In our model, safe and equitable care is the goal for the provision of all end-of-life care services, thus represented as the ‘roof’ of the house of care. Our findings show that safety has a distinct meaning at the end of life. In the literature, patient safety is commonly described as ‘a framework of organised activities [to deliver] consistently and sustainably lower risks’.^20^ In our study, family carers predominantly (though not exclusively) described aspects of the concept of psychological safety, underpinned by timely receipt of care, clear and honest communication, and kind and empathic care delivery. Previous studies have shown that feelings of security are an important component of home-based palliative care^21^ and that feeling unsafe is a common driver of seeking emergency care.^22^ In our data, the relational aspects of how care is provided pervaded the whole experience and particularly impacted on perceptions of quality and safety of care. Thus, the nature of communication and care envelopes all other aspects of care quality.

### Strengths and limitations

The free-text responses were from a national survey (using stratified sampling by gender, causes of death, place of death, age and geographical area), and incorporated data from over 1000 bereaved family members, making it the largest post-bereavement survey in the UK since the 2015 VOICES survey.^14^ The sample reflects the people who died in England and Wales in terms of gender, age, cause of death and place of death. There were slightly more responses from South-East England and South-West Wales, and from those who described their relative as financially more well off.^13^ Importantly, we capture insights into the quality of end-of-life care following the Covid-19 pandemic, during which time there has been a sustained increase in home deaths, widening of inequalities, and changes to health and care delivery.^23^ Previous research on quality of end-of-life care has relied on professionals’ views^3^ or measured structural inputs such as funding and policies as proxies for quality.^24^ The family carer perspective is a strength, as family carers are well placed to judge quality of care.^25^ The involvement throughout this research of people with experience of advanced illness and caring across a range of settings helped ensure its focus and relevance, and their involvement with data analysis contributed to the credibility of the findings. A limitation of our findings is that responses were about the last 3 months of life and often did not specify if they related to one or all settings. Findings from closed questions with analysis by setting are published elsewhere.^13^ However, the variation in descriptions of care, in a range of settings, means that the themes we have constructed are relevant across settings.^26^ While the free-text responses provided were often substantial in length, opportunities to explore and elucidate issues raised were not possible as would have been the case for an interview study.

### Recommendations for policy, practice and research

The context of health care is changing rapidly, due to ageing populations with multiple conditions resulting in an unprecedented demand for care. The need for palliative care in England and Wales is projected to increase 25%–40% by 2040.^27^ Our data identify stark deficiencies in the quality of end-of-life care that must be addressed, and our model of quality of end-of-life care provides a novel approach to conceptualising and guiding improvement.

This model can guide local and national policy makers to develop and monitor end-of-life care, with a focus on safety (both physical and psychological) and equity (see Box 1 for policy and practice recommendations). The model can also support commissioners in measuring and assuring the quality of end-of-life care,^15^ by identifying the critical elements of access, coordination and relational care.

## Box 1. Policy and practice recommendations

Policy and practice recommendations

**Table:** 

1. Increase resources for care services, especially in primary care, to address the issues described by bereaved families and to address health, social care and specialist palliative care workforce training, recruitment, retention and psychological support
2. Improve coordination and timeliness of services, including consideration of one point of contact and dedicated palliative care telephone lines
3. Support better communication and relational care through advanced communication training for all professionals caring for people approaching the end of life, including in hospital settings
4. Invest in services to support family carers in the community, for example with telephone advice, respite and night sitting services, and with bereavement services after the death of their loved one
5. Improve public understanding of end-of-life care to better prepare people for engaging with dying, death, and loss, to build resilience and community capacity
6. Strengthen capacity of local communities to support end-of-life care, through provision of public information resources outlining what is available and how that can be accessed
7. Use the proposed model of quality for end-of-life care to inform commissioning and identification of appropriate performance indicators, including emotional and physical safety, communication and relational care

In addition to financial costs for family carers,^25^ the emotional and physical toll on family carers presents important challenges for health and social care provision and bereavement support.^28^ Initiatives to enhance support for family carers, including navigating an unfamiliar system, should be explored including public health approaches such as strengthening capacity of local communities and resilience for engaging with dying, death and bereavement.^29^

## Conclusion

Ensuring high quality care for people approaching the end of life is a public and policy priority, which has been magnified by the effects of the Covid-19 pandemic, demographic changes and increase in people dying with palliative care needs. Our study finds that many bereaved family members describe care towards the end of life as poor, with access, timeliness and coordination identified as obstacles for high quality care. This impacts on dying people’s quality-of-life and death as well as their families’ physical, emotional and financial wellbeing. Our study identifies key aspects of the quality of end-of-life care that should be the focus for improvement, and provides a refined model of quality for end-of-life care to guide future policy and research.

## Supplemental Material

Supplemental Material - What is the quality of care at the end of life? Qualitative findings from a nationally-representative post-bereavement survey across England and Wales Supplemental Material for What is the quality of care at the end of life? Qualitative findings from a nationally-representative post-bereavement survey across England and Wales by Joanna Goodrich, Sophie Pask, Chukwuebuka Okwuosa, Therese Johansson, Lynn Laidlaw, Cara Ghiglieri, Rachel Chambers, Anna E. Bone, Stephen Barclay, Fliss E. M. Murtagh and Katherine E. Sleeman in Journal of Health Services Research & Policy
