# Supplementary material for: What is the quality of care at the end of life? Qualitative findings from a nationally-representative post-bereavement survey across England and Wales
Source: J Health Serv Res Policy. 2025 Dec 29;31(2):85–97. doi: 10.1177/13558196251398678 (PMC12988001; doi:10.1177/13558196251398678)
Supplement: Supplemental Material - What is the quality of care at the end of life? Qualitative findings from a nationally-representative post-bereavement survey across England and Wales [file sj-pdf-1-hsr-10.1177_13558196251398678.pdf]

Journal of Health Services Research and Policy
What is the quality of care at the end of life? Qualitative findings from a nationally representative post-bereavement survey across England and Wales.
Fliss EM Murtagh et al.

Table S1: Sampling frame

**Variable**	**Description**
Gender	Male; female
Age	Age groups 18-44; 45-64; 65-74; 75-84; 85+
Cause of death	Non-sudden deaths from the following causes: (Malignant neoplasm (ICD codes C00-C97), Heart disease (I00-I52, I60-I69), Renal disease (N17, N18, N28), Liver disease (K70-K77), Respiratory disease (J06-J18, J20-J22, J40-J47, J69), Neurological disease (G10, G12.2, G20, G23.1, G35, G90.3), Dementia (F01, F03, G30, R54), HIV/AIDS (B20-B24)
Place of death	Home, hospital, care home, hospice, other
Geographical area	All areas of England and Wales

Journal of Health Services Research and Policy
What is the quality of care at the end of life? Qualitative findings from a nationally representative post-bereavement survey across England and Wales.
Fliss EM Murtagh et al.

Table S2: Demographic characteristics of decedents

**By response** / **Demographic characteristic**	**For all responses (*N* = 1194)**	**For free-text responses (*N* = 1083)**	**For no free-text responses (*N* = 111)**
**Age at death**			
Mean (SD)	81.5 (11.7)	81.4 (11.7)	82.2 (12.4)
Missing *n* (%)	13 (1.1)	11 (1.0)	2 (1.8)
**Gender** *n* (%)			
Female	615 (51.5)	566 (52.3)	49 (44.1)
Male	571 (47.8)	510 (47.1)	61 (55.0)
Missing	8 (0.7)	7 (0.6)	1 (0.9)
**Ethnicity** *n* (%)			
White British	1142 (95.6)	1036 (95.7)	106 (95.5)
White other	21 (1.8)	19 (1.8)	*
Asian	14 (1.2)	11 (1.0)	*
Black	5 (0.4)	5 (0.5)	-
Any other ethnic group	5 (0.4)	5 (0.5)	-
Mixed	3 (0.3)	3 (0.3)	-
Missing	4 (0.3)	4 (0.2)	-
**Nationality** *n* (%)			
England	611 (51.2)	563 (52.0)	48 (43.2)
Wales	567 (47.5)	505 (46.6)	62 (55.9)
Missing	16 (1.3)	15 (1.4)	1 (0.9)
**Financial circumstance** *n* (%)			
Living comfortably	545 (45.6)	501 (46.3)	44 (39.6)
Doing alright	398 (33.3)	356 (32.9)	42 (37.8)
Just about getting by or finding it difficult	231 (19.3)	208 (19.2)	23 (20.7)
Missing	20 (1.7)	18 (1.6)	2 (1.9)
**Main cause of death** *n* (%)			

Cancer	324 (27.1)	306 (28.3)	18 (16.2)
Multiple diseases	288 (24.1)	261 (24.1)	27 (24.3)
Heart disease	149 (12.5)	133 (12.4)	16 (14.4)
Dementia/ Alzheimer's disease	130 (10.9)	115 (10.6)	15 (13.5)
Lung disease	48 (4.0)	38 (3.5)	10 (9.0)
Stroke	35 (2.9)	33 (3.0)	*
Neurological disease	19 (1.6)	17 (1.6)	*
Liver disease	16 (1.3)	10 (0.9)	6 (5.4)
Kidney disease	5 (0.4)	4 (0.4)	*
Other	165 (13.8)	153 (14.1)	12 (10.8)
Missing	15 (1.3)	13 (1.1)	2 (1.9)
**Location at final 3 months of life** *n* (%)			
Private home (own, family/friend)	739 (61.9)	672 (62.0)	67 (60.4)
Care home	278 (23.3)	255 (23.5)	23 (20.7)
Hospital	118 (9.9)	103 (9.5)	15 (13.5)
Hospice	7 (0.6)	5 (0.5)	*
Other (elsewhere, multiple)	43 (3.6)	39 (3.6)	*
Missing	9 (0.8)	9 (0.9)	-
**Place of death** *n* (%)			
In a hospital	448 (37.5)	405 (37.4)	43 (38.7)
In their, relatives or friends' home	377 (31.6)	348 (32.1)	29 (26.1)
In a care home	282 (23.6)	258 (23.8)	24 (21.6)
In a hospice	63 (5.3)	52 (4.8)	11 (9.9)
Elsewhere	11 (0.9)	11 (1.0)	-
Missing	13 (1.1)	9 (0.9)	4 (3.7)

** denotes suppressed data (cell counts <3)*

Journal of Health Services Research and Policy
What is the quality of care at the end of life? Qualitative findings from a nationally representative post-bereavement survey across England and Wales.
Fliss EM Murtagh et al.

Supplement 1: GRIPP 2

Patient and public involvement with this study

Table S1: GRIPP2 reporting checklist (short form)

Section and topic	Item	Reported on page No
1: Aim	Report the aim of PPI in the study	
2: Methods	Provide a clear description of the methods used for PPI in the study	
3: Study results	Outcomes—Report the results of PPI in the study, including both positive and negative outcomes	
4: Discussion and conclusions	Outcomes—Comment on the extent to which PPI influenced the study overall. Describe positive and negative effects	
5: Reflections/critical perspective	Comment critically on the study, reflecting on the things that went well and those that did not, so others can learn from this experience	

Reference: Staniszewska S, Brett J, Simera I, et al. GRIPP2 reporting checklists: tools to improve reporting of patient and public involvement in research. *BMJ* 2017; j3453

Aim

The data in this paper are from the survey conducted as part of the Marie Curie funded Better End of Life programme. A PPI group was recruited from the 4 nations of the UK and comprised people who had experience of caring for family at the end of life and/or were living with life-limiting illness.

The PPI group has been involved during the whole project with any aspects of the research they wanted closer involvement with. The aim of PPI in this project was to:

1) Advise on the survey including language used in questions and accompanying study information, and providing options for how participants could complete it.
2) Undertake, with the research team, a collaborative, thematic analysis of the free text responses involving differing positionalities and perspectives
3) Advise on the dissemination of research findings.

Methods

A mix of methods were used to incorporate PPI. Meetings were held with the PPI group to discuss survey results and interpretation of findings. 2 members, CG and LL, were involved in the thematic analysis, including coding of the free-text comments and discussions to refine themes. These discussions were reflexive and acknowledged the different perspectives offered.

All members of the PPI group were invited to comment on the first draft of this paper. CG and LL provided in-depth comments and suggested changes to the presentations of findings.

We built trusting relationships with each other which have continued into other research projects.

We valued equally the different types of knowledge and expertise that people had.

PPI members suggested completing the GRIPP 2 checklist to accompany this paper. LL wrote the first draft and it was circulated it to interested members of the team to add comments and suggestions.

Study results

People were offered payment for their time at NIHR rates, meetings were well facilitated, organised in advance and notes were circulated afterwards.

The survey generated a large amount of free-text data and it felt overwhelming at times trying to analyse this and develop themes. The PPI members felt a great responsibility to "get it right" and ensure we were honouring and understanding the experiences of the respondents. The collaborative analysis was a strength but there was a large amount of emotional labour involved for everyone.

The research team were supportive and we felt like part of the team. CD and LL have experience of qualitative research so were able to hit the ground running. The analysis meetings were both enjoyable and challenging, we all learned from each other.

Discussion and conclusions

Involving people with lived experience of caring for family members at the end of life and of life-limiting illness added to the interpretation of the data. Involving different positionalities and perspectives in qualitative analysis can be challenging and time consuming, it requires resources and the will to work in this way.

There is no doubt that it is challenging to embrace the "productive tensions", be reflexive and understand that everyone in the team is coming from a different positionality and perspective. Through building theserelationships we developed respect for multiple opinions.

Reflections/ critical perspective

Involving PPI contributors in thematic analysis of free-text survey responses is possible if the research team value working in this way. Different types of knowledge and expertise need to be respected and there must be resources allocated to support the process. The emotional labour of research into end-of-life care should not be underestimated. Building trusting relationships, supporting each other, and respecting different perspectives and reaching compromise where needed is essential.

Journal of Health Services Research and Policy
What is the quality of care at the end of life? Qualitative findings from a nationally representative post-bereavement survey across England and Wales.
Fliss EM Murtagh et al.

Supplement 2: Checklist for Reporting Of Survey Studies (CROSS)

Section/topic	Item	Item description	Reported on page #
**Title and abstract**			
Title and abstract	1a	State the word "survey" along with a commonly used term in title or abstract to introduce the study's design.	1
	1b	Provide an informative summary in the abstract, covering background, objectives, methods, findings/results, interpretation/discussion, and conclusions.	2
**Introduction**			
Background	2	Provide a background about the rationale of study, what has been previously done, and why this survey is needed.	5-6
Purpose/aim	3	Identify specific purposes, aims, goals, or objectives of the study.	6
**Methods**			
Study design	4	Specify the study design in the methods section with a commonly used term (e.g., cross-sectional or longitudinal).	6
Data collection methods	5a	Describe the questionnaire (e.g., number of sections, number of questions, number and names of instruments used).	Supplementary files 1 and 5
	5b	Describe all questionnaire instruments that were used in the survey to measure particular concepts. Report target population, reported validity and reliability information, scoring/classification procedure, and reference links (if any).	6
	5c	Provide information on pretesting of the questionnaire, if performed (in the article or in an online supplement). Report the method of pretesting, number of times questionnaire was pre-tested, number and demographics of participants used for pretesting, and the level of similarity of demographics between pre-testing participants and sample population.	Not performed
	5d	Questionnaire if possible, should be fully provided (in the article, or as appendices or as an online supplement).	Supplement 1
Sample characteristics	6a	Describe the study population (i.e., background, locations, eligibility criteria for participant inclusion in survey, exclusion criteria).	6-7
	6b	Describe the sampling techniques used (e.g., single stage or multistage sampling, simple random sampling, stratified sampling, cluster sampling, convenience sampling). Specify the locations of sample participants whenever clustered sampling was applied.	7 and supplementary file 5, p.68
	6c	Provide information on sample size, along with details of sample size calculation.	7 and protocol (supplementary file 4)

	6d	Describe how representative the sample is of the study population (or target population if possible), particularly for population-based surveys.	Protocol (supplementary file 4)
Survey administration	7a	Provide information on modes of questionnaire administration, including the type and number of contacts, the location where the survey was conducted (e.g., outpatient room or by use of online tools, such as SurveyMonkey).	8
	7b	Provide information of survey's time frame, such as periods of recruitment, exposure, and follow-up days.	8
	7c	Provide information on the entry process:<br>–>For non-web-based surveys, provide approaches to minimize human error in data entry.<br>–>For web-based surveys, provide approaches to prevent "multiple participation" of participants.	9<br>Protocol (supplementary file 4)
Study preparation	8	Describe any preparation process before conducting the survey (e.g., interviewers' training process, advertising the survey).	Not applicable
Ethical considerations	9a	Provide information on ethical approval for the survey if obtained, including informed consent, institutional review board [IRB] approval, Helsinki declaration, and good clinical practice [GCP] declaration (as appropriate).	9
	9b	Provide information about survey anonymity and confidentiality and describe what mechanisms were used to protect unauthorized access.	10
Statistical analysis	10a	Describe statistical methods and analytical approach. Report the statistical software that was used for data analysis.	9
	10b	Report any modification of variables used in the analysis, along with reference (if available).	n/a
	10c	Report details about how missing data was handled. Include rate of missing items missing data mechanism (i.e., missing completely at random [MCAR], missing at random [MAR] or missing not at random [MNAR]) and methods used to deal with missing data (e.g., multiple imputation).	Not applicable for this analysis
	10d	State how non-response error was addressed.	Not applicable for this analysis
	10e	For longitudinal surveys, state how loss to follow-up was addressed.	Not applicable

			for this analysis
	10f	Indicate whether any methods such as weighting of items or propensity scores have been used to adjust for non-representativeness of the sample.	Not applicable for this analysis
	10g	Describe any sensitivity analysis conducted.	Not applicable for this analysis
**Results**			
Respondent characteristics	11a	Report numbers of individuals at each stage of the study. Consider using a flow diagram, if possible.	
	11b	Provide reasons for non-participation at each stage, if possible.	
	11c	Report response rate, present the definition of response rate or the formula used to calculate response rate.	10 and suppleme ntary file 5, appdx 1
	11d	Provide information to define how unique visitors are determined. Report number of unique visitors along with relevant proportions (e.g., view proportion, participation proportion, completion proportion).	
Descriptive results	12	Provide characteristics of study participants, as well as information on potential confounders and assessed outcomes.	Suppleme nt 2
Main findings	13a	Give unadjusted estimates and, if applicable, confounder-adjusted estimates along with 95% confidence intervals and p-values.	Not applicable for this analysis
	13b	For multivariable analysis, provide information on the model building process, model fit statistics, and model assumptions (as appropriate).	Not applicable for this analysis
	13c	Provide details about any sensitivity analysis performed. If there are considerable amount of missing data, report sensitivity analyses comparing the results of complete cases with that of the imputed dataset (if possible).	Not applicable for this analysis
**Discussion**			
Limitations	14	Discuss the limitations of the study, considering sources of potential biases and imprecisions, such as non-representativeness of sample, study design, important uncontrolled confounders.	34-35
Interpretations	15	Give a cautious overall interpretation of results, based on potential biases and	35-36

		imprecisions and suggest areas for future research.	
Generalizability	16	Discuss the external validity of the results.	35
**Other sections**			
Role of funding source	17	State whether any funding organization has had any roles in the survey's design, implementation, and analysis.	38
Conflict of interest	18	Declare any potential conflict of interest.	38
Acknowledgements	19	Provide names of organizations/persons that are acknowledged along with their contribution to the research.	38,39

Supplement 3: Consolidated criteria for reporting qualitative studies (COREQ): 32-item checklist

No. Item	Guide questions/description	Reported on Page #
**Domain 1: Research team and reflexivity**		
Personal Characteristics		
1. Interviewer/facilitator	Which author/s conducted the interview or focus group?	n/a there were no interviews (this was analysis of freetext questions on survey, p.5)
2. Credentials	What were the researcher's credentials? E.g. PhD, MD	n/a
3. Occupation	What was their occupation at the time of the study?	n/a
4. Gender	Was the researcher male or female?	n/a
5. Experience and training	What experience or training did the researcher have?	n/a
Relationship with participants		
6. Relationship established	Was a relationship established prior to study commencement?	n/a
7. Participant knowledge of the interviewer	What did the participants know about the researcher? e.g. personal goals, reasons for doing the research	n/a
8. Interviewer characteristics	What characteristics were reported about the interviewer/facilitator? e.g. Bias, assumptions, reasons and interests in the research topic	n/a

**Domain 2: study design**		
Theoretical framework		
9. Methodological orientation and Theory	What methodological orientation was stated to underpin the study? e.g. grounded theory, discourse analysis, ethnography, phenomenology, content analysis	5
Participant selection		
10. Sampling	How were participants selected? e.g. purposive, convenience, consecutive, snowball	5
11. Method of approach	How were participants approached? e.g. face-to-face, telephone, mail, email	5
12. Sample size	How many participants were in the study?	5, 7
13. Non-participation	How many people refused to participate or dropped out? Reasons?	5
Setting		
14. Setting of data collection	Where was the data collected? e.g. home, clinic, workplace	7
15. Presence of non-participants	Was anyone else present besides the participants and researchers?	n/a
16. Description of sample	What are the important characteristics of the sample? e.g. demographic data, date	Table 2
Data collection		
17. Interview guide	Were questions, prompts, guides provided by the authors? Was it pilot tested?	Survey questions provided as supplementary file 1
18. Repeat interviews	Were repeat interviews carried out? If yes, how many?	n/a
19. Audio/visual recording	Did the research use audio or visual recording to collect the data?	n/a
20. Field notes	Were field notes made during and/or after the interview or focus group?	n/a
21. Duration	What was the duration of the interviews or focus group?	n/a
22. Data saturation	Was data saturation discussed?	n/a
23. Transcripts returned	Were transcripts returned to participants for comment and/or correction?	n/a
**Domain 3: analysis and findings**		
Data analysis		

24. Number of data coders	How many data coders coded the data?	5,6 three researchers and two PPI partners
25. Description of the coding tree	Did authors provide a description of the coding tree?	no
26. Derivation of themes	Were themes identified in advance or derived from the data?	5,6
27. Software	What software, if applicable, was used to manage the data?	6
28. Participant checking	Did participants provide feedback on the findings?	6<br>PPI partners did
Reporting		
29. Quotations presented	Were participant quotations presented to illustrate the themes/findings? Was each quotation identified? e.g. participant number	10-26
30. Data and findings consistent	Was there consistency between the data presented and the findings?	10-26
31. Clarity of major themes	Were major themes clearly presented in the findings?	10-26
32. Clarity of minor themes	Is there a description of diverse cases or discussion of minor themes?	no
